# Supplementary material for: Self-management of chronic, non-communicable diseases in South Asian settings: A systematic mixed-studies review
Source: PLOS Glob Public Health. 2024 Jan 8;4(1):e0001668. doi: 10.1371/journal.pgph.0001668 (PMC10773968; doi:10.1371/journal.pgph.0001668)
Supplement: S2 Table — (DOCX) [file pgph.0001668.s002.docx]

S2 Table. Item-specific and overall quality scoring of primary quantitative, qualitative and mixed-methods studies using the MMAT checklist (n=44)

| **Quantitative studies (n=26)** | | | | | | |
| --- | --- | --- | --- | --- | --- | --- |
| **Author, year** | **1.1. Representativeness of study sample** | **1.2. Appropriateness of measurement methods** | **1.3. Completeness of outcome data** | **1.4. Adjustment for confounders** | **1.5. Change in exposure status** | **Overall quality score** |
| **Aggarwal, 2020** | can't tell | yes | yes | can't tell | not applicable | 2 |
| **Ajani, 2021** | can't tell | can't tell | yes | can't tell | not applicable | 1 |
| **Arulmozhi, 2014** | can't tell | can't tell | yes | can't tell | not applicable | 1 |
| **Basu, 2018** | can't tell | can't tell | yes | can't tell | not applicable | 1 |
| **Bhandari B, 2015** | yes | can't tell | yes | can't tell | not applicable | 2 |
| **Bhandari S, 2015** | yes | can't tell | yes | can't tell | not applicable | 2 |
| **Chandrika, 2020** | yes | can't tell | can't tell | can't tell | not applicable | 1 |
| **Ghimire, 2017** | no | no | yes | yes | not applicable | 2 |
| **Ghimire, 2018** | can't tell | can't tell | yes | yes | not applicable | 2 |
| **Gopichandran, 2012** | yes | can't tell | yes | can't tell | not applicable | 2 |
| **Gowani, 2017 (a)** | yes | can't tell | yes | can't tell | not applicable | 2 |
| **Gupta, 2022** | yes | yes | yes | can’t tell | not applicable | 3 |
| **Kandel, 2022** | yes | can’t tell | yes | yes | not applicable | 3 |
| **Khanam, 2014** | yes | can't tell | can't tell | can't tell | not applicable | 1 |
| **Koirala, 2020** | can't tell | yes | yes | yes | not applicable | 3 |
| **Mahmood, 2020** | yes | yes | yes | can't tell | not applicable | 3 |
| **Mannan, 2021** | yes | yes | yes | can't tell | not applicable | 3 |
| **Rafi, 2022** | can't tell | no | can't tell | can't tell | not applicable | 0 |
| **Rao, 2014** | yes | can't tell | yes | can't tell | not applicable | 2 |
| **Ravi, 2018** | can't tell | can't tell | yes | yes | not applicable | 2 |
| **Roka, 2019** | can't tell | can't tell | yes | yes | not applicable | 2 |
| **Shani, 2021** | yes | can't tell | yes | yes | not applicable | 3 |
| **Shrestha, 2021** | yes | yes | yes | yes | not applicable | 4 |
| **Srirari, 2019** | yes | can't tell | can't tell | can't tell | not applicable | 1 |
| **Saqlain, 2019** | can't tell | can't tell | yes | can't tell | not applicable | 1 |
| **Yadav, 2020 (a)** | Yes | can't tell | yes | can't tell | not applicable | 2 |
| **Qualitative studies (n= 16)** | | | | | | |
| **Author, year** | **1.1. Appropriate use of qualitative approach** | **1.2. Adequate data collection methods used** | **1.3. Findings adequately derived from data** | **1.4. Results sufficiently substantiated by data** | **1.5. Coherence between data sources, data collection, analysis and interpretation** | **Overall quality score** |
| **Adhikari, 2021** | yes | yes | can't tell | can't tell | yes | 3 |
| **Anitha Rani, 2019** | yes | can't tell | can't tell | yes | yes | 3 |
| **Ansari, 2021** | yes | can't tell | can't tell | yes | yes | 3 |
| **Ansari, 2019** | yes | can't tell | can't tell | yes | can't tell | 2 |
| **Basu, 2020** | yes | can't tell | yes | yes | yes | 4 |
| **Buksh, 2020** | yes | yes | yes | yes | yes | 5 |
| **Chittem, 2021** | yes | can't tell | can't tell | yes | yes | 3 |
| **Gowani, 2017 (b)** | yes | yes | can't tell | yes | can't tell | 3 |
| **Gupta, 2019** | can't tell | can't tell | can't tell | can't tell | can't tell | 0 |
| **Islam, 2017** | yes | yes | yes | yes | no | 4 |
| **Jose, 2020** | yes | can't tell | yes | can't tell | yes | 3 |
| **Kamath, 2020** | yes | yes | yes | yes | yes | 5 |
| **Kamath, 2021** | yes | yes | yes | yes | yes | 5 |
| **Matpady, 2020** | yes | yes | can't tell | can't tell | yes | 3 |
| **Yadav, 2020 (b)** | yes | yes | can't tell | yes | yes | 4 |
| **Zeb, 2020** | yes | yes | yes | yes | yes | 5 |
| Mixed-Methods studies (n=2) | | | | | | |
| **Author, year** | **1.1. Adequate rationale for mixed-methods design** | **1.2. Effective integration of QT/QL study components** | **1.3. Effective interpretation of the outputs of integration** | **1.4. Divergences and inconsistencies adequately addressed** | **1.5. Adherence to quality criteria of each (QT/QL) tradition** | **Overall quality score** |
| **Bhandari, P** | yes | yes | yes | yes | can’t tell | 4 |
| **Jennings H, 2021** | yes | yes | yes | yes | can’t tell | 4 |
