# Supplementary material for: Self-management of chronic, non-communicable diseases in South Asian settings: A systematic mixed-studies review
Source: PLOS Glob Public Health. 2024 Jan 8;4(1):e0001668. doi: 10.1371/journal.pgph.0001668 (PMC10773968; doi:10.1371/journal.pgph.0001668)
Supplement: S3 Table — (DOCX) [file pgph.0001668.s003.docx]

S3 Table. List of descriptive themes, sub-themes and individual codes generated in the qualitative thematic synthesis

|  | **Descriptive themes** | **Sub-themes** | **Codes** |
| --- | --- | --- | --- |
| 1. | Self-management is restricted in financial adversity. | Having limited financial means | Insufficient funds to manage illness [(32,36,38,43,47–49,52)](https://paperpile.com/c/yPCG7p/XzpG+Rjza+cRgo+nylMZ+R7QD+EqDy+WsrS+cQn4)  Not having enough financial means to procure items essential for self-management  Not having financial independence [(40,48)](https://paperpile.com/c/yPCG7p/oS8j+cQn4)  Living in retirement, or being unemployed with lack of resources to self-manage |
|  |  | Costs related to self-management | Cost of travelling to seek care [(32,38,43,45,47,48)](https://paperpile.com/c/yPCG7p/XzpG+R7QD+EqDy+WsrS+nRCe+cQn4)  High costs of transport, and forgone income if one seeks the HCP  Costs of healthy foods [(38,40,45,47,49,51)](https://paperpile.com/c/yPCG7p/XzpG+R76C+cRgo+oS8j+EqDy+nRCe)  Having to suffice with the bare minimum, not having sufficient resources to procure fruits/vegs  Costs related to medicine or equipment  [(32,38,39,44,47–50)](https://paperpile.com/c/yPCG7p/XzpG+cRgo+EASw+EqDy+0tTL+WsrS+cQn4+bgOv)  Cost of doctor’s consultations [(38,43,48,52)](https://paperpile.com/c/yPCG7p/XzpG+Rjza+R7QD+cQn4)  Describing money as a barrier to seeking care from HCPs, and carry our required SM tasks |
|  |  | Cost related impacts on self-management | Delaying, or forgoing self-management  Not feeling the need to undergo routine health checks, doctor visits in the absence of an emergency. Either unwarranted or seen as an additional cost.  Seeking subsidised or free medications  Limited contact with the health system, focused primarily on seeking free or low-cost treatments from pharmacies and hospitals. Switching of care providers as a result impacted on continuity of care. |
|  |  |  |  |
| 2. | Support from family and friends: the social network | Receiving personalised support  Receiving encouragement and motivation | Being supported financially [(38,43,44,47,50)](https://paperpile.com/c/yPCG7p/XzpG+R7QD+EqDy+0tTL+bgOv)  Receiving assistance in procuring medications and other financial needs to enable self-management  Making adjustments [(38,43,44,53)](https://paperpile.com/c/yPCG7p/0tTL+R7QD+XzpG+dVNT)  Instances where the family modified their diet, to suit needs of the patient  Direct support or assistance [(39–41,43,45,47,49,50)](https://paperpile.com/c/yPCG7p/cRgo+oS8j+EASw+R7QD+EqDy+bgOv+nRCe+tEB1)  Being supported in taking medication, or regular monitoring of illness  Recieving reminders [(38,40,43,45,47,48,50,51,53)](https://paperpile.com/c/yPCG7p/XzpG+R76C+oS8j+R7QD+EqDy+bgOv+nRCe+dVNT+cQn4)  Overseeing that family members with NCDs carry out required self-management tasks on time  Encouragement and moral support [(38,40,41,45,48,50,52)](https://paperpile.com/c/yPCG7p/XzpG+Rjza+oS8j+bgOv+nRCe+tEB1+cQn4)  Sharing illness experiences [(38)](https://paperpile.com/c/yPCG7p/XzpG) |
|  |  |  |  |
|  |  | Negative perceptions and experiences of support | Being a burden on others [(36,39,45,48)](https://paperpile.com/c/yPCG7p/nylMZ+EASw+nRCe+cQn4)  Experiencing neglect or lack of support [(32,38,39,42,44,49,50,52)](https://paperpile.com/c/yPCG7p/XzpG+Rjza+Lvjw+cRgo+EASw+0tTL+WsrS+bgOv)  Having self-management needs overlooked or neglected by those responsible to caregiving |
| 3. | Managing illness at home and the workplace |  | Demanding nature of work [(36,38,40,44,53)](https://paperpile.com/c/yPCG7p/XzpG+nylMZ+oS8j+0tTL+dVNT)  Being overburdened, or fatigued by demands of work  Household responsibilities [(38,40,44,49,51,52)](https://paperpile.com/c/yPCG7p/0tTL+oS8j+cRgo+Rjza+R76C+XzpG)  Being too busy in managing the household to perform self-management. |
| 4. | The role of healthcare providers, and patient-doctor interactions | Healthcare providers’ influences on patient education and self-management | HCPs as a source of information and motivation [(32,38,40,41,45,48,50,53)](https://paperpile.com/c/yPCG7p/XzpG+oS8j+WsrS+bgOv+nRCe+dVNT+tEB1+cQn4)  Provision of self-help materials [(40)](https://paperpile.com/c/yPCG7p/oS8j)  Receiving informational materials around illness management either from the clinic or HCP |
|  |  | Experiences of patient-doctor interactions | Not being guided  [(38,40,41,43–45,47,50–52)](https://paperpile.com/c/yPCG7p/XzpG+R76C+Rjza+oS8j+R7QD+EqDy+0tTL+bgOv+nRCe+tEB1)  Lack of information or support provision from the healthcare provider  provision of incomplete information [(38,40,41,43,44,47,50,51)](https://paperpile.com/c/yPCG7p/XzpG+R76C+oS8j+R7QD+EqDy+0tTL+bgOv+tEB1)  being given inconsistent, conflicting or confusing information regarding illness management  Difficulty in communication [(39,41,47)](https://paperpile.com/c/yPCG7p/EASw+EqDy+tEB1)  Having low confidence or feeling reluctant to discuss personal care with the doctor. |
| 5. | Disease related knowledge and illness management: the role of alternate sources | Sources of information | Lacking information to guide SM  [(38,40,41,43,51–53)](https://paperpile.com/c/yPCG7p/XzpG+R76C+Rjza+oS8j+R7QD+dVNT+tEB1)  Listening to social acquaintances [(32,38–41,45)](https://paperpile.com/c/yPCG7p/XzpG+oS8j+EASw+WsrS+nRCe+tEB1)  Scepticism related to treatment and HCPs [(32,41,45,47,52)](https://paperpile.com/c/yPCG7p/tEB1+nRCe+WsrS+EqDy+Rjza)  Disappointment in treatment, and doubts over its efficacy and intended purpose |
|  |  | Self-directed actions and alternative/complementary treatments | Taking a self-directed approach [(32,38,44,49,51–53)](https://paperpile.com/c/yPCG7p/XzpG+R76C+Rjza+cRgo+0tTL+WsrS+dVNT)  Independently deciding on the approach to self-management activities, based on perceived or observed benefit  prioritising symptom relief  prioritising selected SM behaviours  seeking complimentary or alternative care.  at diagnosis, information sought on easily available and low-cost alternative treatments |
| 6. | Challenges to self-management in the social and physical environment | Social engagements/self-management in public | Reluctance to self-manage in public [(39,44,45,51)](https://paperpile.com/c/yPCG7p/R76C+EASw+0tTL+nRCe)  Feeling ashamed, or embarrassed of practising dietary needs, doing exercise, or taking insulin in front of others  Complying with social norms/attending family and social events [(36,38–40,42,52,53)](https://paperpile.com/c/yPCG7p/XzpG+Rjza+Lvjw+nylMZ+oS8j+EASw+dVNT)  Forgoing dietary or medication needs to avoid upsetting others.  Unavailability of healthy choices, [(36,40,43,47,52)](https://paperpile.com/c/yPCG7p/Rjza+nylMZ+oS8j+R7QD+EqDy)  Inability to pursue healthier food options to manage illness due to lack of, or limited “friendly” options |
|  |  | Challenges in the physical environment | Being restricted by the weather, [(38,40,48)](https://paperpile.com/c/yPCG7p/XzpG+oS8j+cQn4) facing natural elements and seasonal variation  Lack of proper facilities, [(38,42,44,52)](https://paperpile.com/c/yPCG7p/0tTL+Rjza+Lvjw+XzpG)  Being restricted by the unavailability of safe and appropriate physical spaces to exercise  Facing accessibility issues [(32,38,43,45)](https://paperpile.com/c/yPCG7p/nRCe+WsrS+XzpG+R7QD) not having healthcare providers and/or healthcare facilities in physical proximity |
| 7. | The impacts of physical and psychological illness manifestations |  | Having multiple health issues [(40,43,47,49,51)](https://paperpile.com/c/yPCG7p/R76C+cRgo+oS8j+R7QD+EqDy)  Physical or mental constraints imposed on self-management of one condition in by the presence of others  Following multiple treatments [(39,41,43,44,47)](https://paperpile.com/c/yPCG7p/tEB1+0tTL+EqDy+R7QD+EASw)  Facing the struggle to adhere to multiple regimens,  Fear of pain or discomfort [(40,48,49,51)](https://paperpile.com/c/yPCG7p/R76C+cRgo+oS8j+cQn4)  Experiencing discomfort due to symptom severity, or from self-management activity (exercise, finger pricking)  Affective and somatic effects of illness [(36,40,43,44,48)](https://paperpile.com/c/yPCG7p/nylMZ+oS8j+R7QD+cQn4+0tTL)  Experiencing physical or psychological consequences of illness |
| 8. | Personal attitudes distinguish between active and passive self-management. | Passivity in self-management | Forgetting to undertake illness management [(32,39,40,44,47,49,53)](https://paperpile.com/c/yPCG7p/dVNT+WsrS+0tTL+EqDy+EASw+oS8j+cRgo)  Either due to carelessness, or due to busy schedule, or due to memory loss  Lack of motivation or interest [(38,39,41,52)](https://paperpile.com/c/yPCG7p/XzpG+Rjza+EASw+tEB1)  Being unbothered to make necessary changes, demonstrating fatalistic attitudes- seen by HCPs as being due to lack of understanding.  Fatalistic views and opinions [(38,45,52)](https://paperpile.com/c/yPCG7p/XzpG+nRCe+Rjza)  Seeing illness as a pre-determined consequence, letting things take their course and being disengaged from self-management |
|  |  |  |  |
|  |  | Proactive self-management | Desire to be healthy  being intrinsically motivated to maintain a healthy self-image  Fear of illness consequences  Being driven by the anxiety of suffering illness consequences  Responsibility towards own health [(38,45,50)](https://paperpile.com/c/yPCG7p/XzpG+bgOv+nRCe)  Taking a proactive approach [(36,38,40,49,53)](https://paperpile.com/c/yPCG7p/XzpG+cRgo+nylMZ+oS8j+dVNT)  Taking initiatives to find suitable ways of maintaining health in illness. |
