# Supplementary material for: Self-management of chronic, non-communicable diseases in South Asian settings: A systematic mixed-studies review
Source: PLOS Glob Public Health. 2024 Jan 8;4(1):e0001668. doi: 10.1371/journal.pgph.0001668 (PMC10773968; doi:10.1371/journal.pgph.0001668)
Supplement: Attachment — Submitted filename: Response to Reviewers.docx [file pgph.0001668.s005.docx]

**Response to reviewers comments**

| **Reviewer #1:** Thank you for inviting me to peer review the manuscript ‘Self-management of chronic, non-communicable diseases in South Asian settings: A systematic mixed-studies review’. Please find below my comments which I believe if addressed will strengthen this review: | |
| --- | --- |
| Lines 31-33 – Please add key databases searched and also the publication dates considered. | See updated text: “We conducted a mixed-methods systematic review, searching key electronic databases [MEDLINE (1946+), Embase (1974+), PsycInfo (1967+) and CINAHL (EBSCOhost)] in March 2022 (and updated in April 2023) to identify quantitative, qualitative and mixed-methods studies…” |
| Line 77 -need to have a full stop before ‘however’ | The sentence has now been rewritten in response to the comment below. |
| Please be more specific about the context upfront. | Thank you for the comment:  We have now added some text to highlight the specific context of South Asian countries, in relation to health systems, socio-cultural practices and beliefs in the introduction, along with appropriate references cited. See highlighted text below:  “Improving self-management in a group of individuals requires context-specific knowledge. This is particularly relevant for South Asian settings and populations, which are characterised by inefficient and poorly resourced health systems [(Ahmed et al., 2019)](https://paperpile.com/c/JzOI8i/NwDm), deep-rooted socio-cultural practices and traditions [(Siddiqui et al., 2021)](https://paperpile.com/c/JzOI8i/GEuF), as well culturally ingrained beliefs [(Patel et al., 2015)](https://paperpile.com/c/JzOI8i/cNVb) and practices around health [(Sohal et al., 2015)](https://paperpile.com/c/JzOI8i/QScR).”  In the preceding paragraph, we also discuss similarities across South Asian populations in the other contexts (i.e. population density, demographics, disease prevalence etc) which makes undertaking this review so relevant. See text below:  “ South Asia is of particular importance in this regard - it is a densely populated LMIC region with a high prevalence of NCDs, particularly cardiometabolic diseases, respiratory diseases and depression [(Hossain et al., 2020; Shams et al., 2021)](https://paperpile.com/c/JzOI8i/O8f7U+M214o). Underlying populations in this region are similar demographically and share common biological, social and environmental influences that shape illness experiences [(Siegel et al., 2014)](https://paperpile.com/c/JzOI8i/uFckO).” |
| *Lines 93-94 – What is your definition of self-management and what are the specific behaviours (and measures for quantitative/mixed-methods studies) you were interested in?* | *Thanks for your comment. We have used the list of self-management behaviours identified by the American Academy of Diabetes Educators, which has direct relevance for diabetes, as well as the other NCDs that we included in our review.*  *See highlighted text below:*  *“*We used the American Academy of Diabetes Educators’ (AADE) list of self-management behaviours for diabetes, which by extension is also relevant to the other NCDs included in this review. These behaviours include: healthy coping, healthy eating, being active, taking medication, monitoring, reducing risk and problem solving [*(7)*](https://paperpile.com/c/yPCG7p/O1NF)*.* |
| I suggest the authors update their searches given the final search was conducted over a year ago. | Thank you for the suggestion. We have updated the search, identifying 25 additional articles of which 2 (both quantiative studies) were eligible for the review. The PRISMA diagram has been updated, quality assessment done for both studies, and findings included in the narrative synthesis and effect direction plots. The additions/edits have been highlighted accordingly in the marked version of the manuscript.  See highlighted text below:  “The original search was carried out in March 2022 on four electronic databases and updated in April 2023. The following databases were searched: MEDLINE (Ovid, Epub Ahead of Print, In-Process, In-Data-Review & Other Non-Indexed Citations and Daily, 1946+), Embase (Ovid, 1974+), APA PsycInfo (Ovid, 1967+) and CINAHL (EBSCOhost).” |
| Lines 134-135 - Please explain why social-political context (eg. conflict) and physical environment (eg, green spaces) were not considered here. | Thank you for your comment.  We have now clearly identified each of the respective categories representing the structural and intermediary determinants of the CSDH framework.  “We used the World Health Organisation’s Commission on Social Determinants of Health (CSDH) framework to identify determinants that act at the structural level (socio-economic and political context, indicators of socioeconomic position) and the intermediary level (material circumstances, behavioural and biological factors, psychosocial factors, health-system related factors)”  We also wish to clarify that while our aim was to identify determinants under each of these categories, we found published evidence to featured some of the categories heavily, with limited (or no) evidence for others. This has been acknowledged in the limitations section.See highlighted text below:  “We found that the quantitative evidence focuses largely on indicators of socio-economic position, and less on determinants acting at the intermediary levels, particularly psychosocial determinants and those at the health-system level. There is also a paucity of data on determinants that constitute the wider socioeconomic and political context (policy, governance, cultural and societal values etc) which are likely to be similar across South Asian countries.” |
| Line 137 – suggest replacing ‘they’ with ‘these’. | Change has been made. |
| Line 155- suggest replacing 42 ‘titles’ with ‘studies’ | Change has been made. |
| Figure 2 – Please explain how from 14,302 titles and abstracts (deduplicated 14,279+23) you have reached 1,631 for 1st stage screening, what happened to 12,671 titles and abstracts? | Thank you for your comment.  The search has been updated and the presentation of the PRISMA flow diagram has been improved in line with the provided feedback. |
| Did you exclude any of the studies based on quality? If not, why? Did you consider how the inclusion of poor quality studies (eg, 8 poor quality quantitative studies) have impacted the results? | Thanks for your comment.  Our approach for both qualitative and quantitative synthesis was to include eligible studies regardless of study quality. This follows standard convention in systematic reviews, and is in line with the aims of the review, which were to provide a comprehensive, overarching synthesis on the topic. We do however, understand and acknowledge that this could have implications for the review findings, which we have unpacked in the strengths and limitations section of our discussion. Please see the following text:  “Quantitative studies were predominantly small-scale and moderate to poor in quality, with limited internal and external validity. Several of these studies relied on non-validated and subjective patient-reported measures to observe self-management behaviours. While inclusion of these studies enabled us to map a range of determinants, interpretation of some of these may warrant caution, as has been clarified in our narrative synthesis. Qualitative studies rated better overall in quality; despite this, several studies only offered contextually thin data which may have limited the depth, interpretability and generalisability of our findings.”  We have also taken this forward in the recommendations section, highlighting the need for better quality studies using objective, validated measures.  “Future research should emphasise the development, implementation and evaluation of the initiatives identified above, while at the same time expanding the knowledge base around self-management, particularly among individuals living with multiple NCDs using objective, validated measures.” |
| Table 2 - This is a nice table, but suggest reformatting it slightly as not clear for the reader the direction of association between a given determinant and multiple outcomes (2 and more outcomes, what specific outcomes do you refer to here?) | Thanks for your comment.  We understand and acknowledge that while the effect direction plot complements and supports a narrative synthesis, it does has certain limitations which have been picked up in this comment, and have been duly acknowledged in our limitations section:  “We used visual methods (effect direction plots) to supplement narrative synthesis. This approach, while broadly useful, has certain limitations: the plots could only be used to present information on the number (and not the type) of self-management behaviours observed in studies of multiple self-management behaviours. We were also unable to provide information on the direction of association for the individual self-management behaviours in such studies, where the observed associations were discordant.”  The information on which behaviours were observed (in studies of multiple behaviours) is available in Table 1, and details on the observed associations are covered to the extent possible in the narrative synthesis. We hope that this provides reasonable reassurance that the work was performed to the highest standard. |
| Lines 418 – Consider the effects of physical environment and wider political context here. | Thank you for your comment.  We have updated the text in the section on policy, practice and research implications. We have mentioned the broader socio-political context and the effects of physical environment, provided recommendations having demonstrated potential and relevance in the context of South Asian countries, and lastly, alluded to the relevance of involving and engaging with key stakeholders in ensuring the sustainability and long term success of these efforts. See highlighted text below.:  “ Within the wider socio-political context of South Asia, the self-management of high-burden NCDs continues to be shaped by a complex network of factors at the individual, household/societal and health system levels, as well as the physical environment. A multi-component, whole systems approach is therefore needed to address differences in disease risk factors, and to support secondary prevention for improving disease outcomes across the social gradient [(Marmot & Bell, 2019)](https://paperpile.com/c/JzOI8i/hlUW6). Empowerment of patients, caregivers and the wider community through knowledge-sharing and education can improve individual and collective attitudes towards NCDs, given the positive attitudes towards health education among South Asian communities [(Stone et al. 2005)](https://paperpile.com/c/JzOI8i/T3cLC). Low-cost, community-based programs in particular have been shown to improve awareness of, and engagement in self-management behaviours, both in South Asian settings and beyond [(Glenn et al. 2020; Balagopal et al. 2008)](https://paperpile.com/c/JzOI8i/QJcjV+nIZEs). On the other hand, the design and delivery of bespoke behaviour change interventions on the other hand, can target high risk groups such as those with multimorbidities, who are at a disproportionate risk of adverse health outcomes [(Singh et al. 2019)](https://paperpile.com/c/JzOI8i/HiJ6).  Health service delivery in South Asian settings can benefit from communication skills training for healthcare providers, enhanced care coordination, as well as task-shifting initiatives such as the involvement of community health workers [(Jafar et al. 2020; Jeet et al. 2017)](https://paperpile.com/c/JzOI8i/ULfg+Nvcse). Efforts in this direction can contribute towards the delivery of standardised care, improved patient counselling and stronger emphasis on self-management, which are core components of the World Health Organisation’s Package of Essential Non-communicable disease interventions (PEN), [(WHO Package of Essential Noncommunicable (PEN) Disease Interventions for Primary Health Care, 2020)](https://paperpile.com/c/JzOI8i/STfwr). Rural and far flung areas can particularly benefit from digital and tele-health initiatives, with several small studies demonstrating its potential in South Asia [(Hearn et al. 2019)](https://paperpile.com/c/JzOI8i/aWHE). However, such approaches need to consider issues regarding accessibility, which is a recognised problem particularly among women in rural settings [(Paul and Dutta 2023)](https://paperpile.com/c/JzOI8i/JpSp). The involvement of, and engagement with stakeholders in the local context (political actors, health and technology services, end-users) in these initiatives is critical to the sustainability and long-term success of these efforts. |
| Lines 420-422 – Please provide specific strategies on how to do these effectively in South Asian context; these are not homogeneous contexts. |  |
| Lines 424-429 – These are very generic statements; I suggest the authors provide further elaborations on (a) future research directions, and (b) how some of the proposed strategies, eg., implementation of package of essential NCD interventions, and others can be implemented considering global actors, context specific local stakeholders and the political environment. |  |

| **Reviewer #2:** This manuscript provides insight into an important area of mitigating the effects of non-communicable diseases (NCDs) with its focus on self-management strategies among individuals living with chronic NCDs. It fills a gap in NCD research associated with self-management strategies—which undeniably are important when one is working to improve the health of individuals with NCDs. The focus examining the social determinants of health including biological, psychosocial and health system factors and what drives self-care is a strength of this manuscript. | |
| --- | --- |
| This research will serve as the basis for additional qualitative and quantitative research documenting what affects self-care among individuals with NCDs. Further research, as the authors point out should serve as the basis for building programs to mitigate these individuals’ challenges. One of the strengths of this comprehensive review is the inclusion of both qualitative, quantitative, and mixed methods studies. One limitation that ought to be pointed out is the use of self-reported adherence outcomes in most research reviewed—a limitation not of the manuscript—but of the current status of research on self-care—including adherence in the field. This well-written manuscript which needs minor editing for a few typos (e.g. in Figure 2)." | Thank you for your comments.  We have included your point regarding self-report in the strengths and limitations section, and clearly highlighted the need for future research to use more objective, validated measures in the recommendations section. See the text:  Strengths and limitations: “Several of these studies relied on non-validated and subjective patient-reported measures to observe self-management behaviours.  Recommendations: Future research should emphasise the development, implementation and evaluation of the initiatives identified above, while at the same time expanding the knowledge base around self-management, particularly among individuals living with multiple NCDs using objective, validated measures.  We have thoroughly read and re-read the manuscript and tables to ensure all  typos are corrected. |

| **Reviewer #3:** I appreciate the opportunity to provide comments on this systematic review of mixed methods by Siddiqui et al. The review addresses an important gap in the literature to better understand the determinants of NCD self-management in the South Asia region. While I am very enthusiastic about the focus of the review, there are a few questions and concerns that I have about the methodology that make it difficult to understand the presented results. | |
| --- | --- |
| Data availability: There are inconsistencies between the provided statement “All the data underlying our findings are included within the manuscript and supplementary information provided.” and the data in the supplementary materials. For example, the ‘final search strategies’ include ‘Date of search: 1st March 2021’. This information does not align with the presented information in the methodology of the main text. Please check that all data are presented accurately. | Thank you for your comment. Based on the reviewers’ comments, we have now updated the search strategy document and checked the supplementary tables, adding in clarification and removing any inconsistencies identified. This has also been clarified in our response to reviewer 1 (see above) |
| In lines 85 – 86 the registered PROSPERO (CRD42021240899) does not appear to match the title and information presented in this manuscript. It appears that the search strategy casted a very wide net to look at many different countries that were not just ‘South Asian’ and it would be helpful to know why the search was conducted in this way. | Thank you for highlighting this discrepancy. We initially developed this review to consolidate evidence for LMICs but the protocol was later amended to focus on South Asian countries specifically, while keeping the searches broad to gauge the amount of evidence published from South Asian countries within the wider umbrella of LMICs. We regret that the amendment was not reflected in the registered protocol at the time of submitting this manuscript, but we have now made the relevant updates in PROSPERO to provide a more accurate description of the protocol. |
| In lines 105-107 Did you identify any additional studies from the grey literature search and citation chaining? This is not clear in the text, nor in the PRISMA flow chart.  Please be consistent with your language used throughout the manuscript. For example in line 188, ‘indicators of socioeconomic position’ is mentioned first and then in line 203 ‘SES indices’. These are two distinct concepts and should be clarified. | Thank you for your comment. We have updated the PRISMA flow diagram for clarity and updated the text in the opening results section: “A total of 22,112 titles were identified through electronic database searches (n=22,089) and searches of reference and grey literature (n=23).”  Regarding the second point, we agree that these are different concepts (SES indices are used to measure and indicate the socio-economic position). We have made this clearer in the text where it was originally mentioned (line **____:)** “..measured using SES indices, self-reported assets, and household and family income..” |
| While I appreciate the conceptual map of self-management in Figure 2, it is difficult for me to see how this is designed specifically for the South Asian context – what about racism? | Thank you for the comment. This review was designed and conducted with a specific population/region (South Asia) in mind. The conceptual map presented in Figure 2 provides a visual representation of the findings of our syntheses. It is therefore not designed for, but rather informed by the studies conducted in South Asia. We also wish to clarify here that while the figure illustrates pathways that influence self-management behaviours in the South Asian context, this is by no means limited to South Asian settings. As we allude to in our discussion section, several of these findings draw parallels with evidence from wider LMIC settings. |
| In lines 396 – 397 : considering that “the number of South Asian studies on self-management has grown considerably in the past decade”, it would be important to acknowledge when the literature search was conducted and using the World Bank ‘South Asia’ region definition for screening as limitation of this work. Whether the search was conducted in March 2022 (as stated in the abstract) or March 2021 (in the supplementary materials), this context is necessary to understand to improve the interpretation of the findings. | Thank you for your comment.  We wish to clarify that we purposely used the World Bank list for South Asian countries, as we found it to be the most relevant and comprehensive list of regional countries (8 countries in total). This in our view, is a strength of how we conducted the review (given the focus was South Asia itself) rather than a limitation.  Regarding the second point, we have updated the supplementary file to indicate the search being conducted in March 2022 (and updated in April 2023, in line with recommendations of reviewer 1). The text in the main manuscript is consistent with this change. |
| **Minor:**  Every table, even in the supplementary materials, should be able to stand-alone. Please check and make sure that all necessary information to understand the data in tables are shown as footnotes. For example, “SMSR ED plots” and Table 2 do not define what the acronyms SM and MA mean. It would also be helpful to remind readers what the points mean and how they were calculated to interpret the table data. | Thank you for your comment. We have reviewed all the tables to ensure they provide sufficient detail and clarity to stand-alone. The typos and omissions in the SMSR ED plot and table 2 have been addressed. |
