# Supplementary material for: Self-management of chronic, non-communicable diseases in South Asian settings: A systematic mixed-studies review
Source: PLOS Glob Public Health. 2024 Jan 8;4(1):e0001668. doi: 10.1371/journal.pgph.0001668 (PMC10773968; doi:10.1371/journal.pgph.0001668)
Supplement: Attachment — Submitted filename: Response to reviewers.docx [file pgph.0001668.s006.docx]

**Response to reviewers comments**

| **Journal Requirements:** | |
| --- | --- |
| 1. Please review your reference list to ensure that it is complete and correct. If you have cited papers that have been retracted, please include the rationale for doing so in the manuscript text, or remove these references and replace them with relevant current references. Any changes to the reference list should be mentioned in the rebuttal letter that accompanies your revised manuscript. If you need to cite a retracted article, indicate the article’s retracted status in the References list and also include a citation and full reference for the retraction notice. | We have carefully reviewed our reference list and cross-checked it using the retraction watch database (retractiondatabase.org). To the best of our knowledge, our reference list does not contain any retracted papers. |
| 2. Please ensure that Funding Information and Financial Disclosure Statement are matched. | Thank you for pointing this out. The funding information has now been updated in line with the journal requirements.  Three of the authors are Principal/co-investigators in the DiaDeM global health research program, which was the source of funding for the primary author. The declaration of interests statement has been updated to reflect this. |
| 3. In the Funding Information you indicated that no funding was received. Please revise the Funding Information field to reflect funding received. |  |
| 4. We do not publish any copyright or trademark symbols that usually accompany proprietary names, eg ©, ®, ™ (e.g. next to drug or reagent names). Please remove all instances of trademark/copyright symbols throughout the text, including ® on page 30. | We have removed the ® on page 30,and carefully reviewed the manuscript to exclude any such symbols. |
| **Comments to the Author:** | |
| Reviewer #3: Thank you for the opportunity to re-review the manuscript. The manuscript describes factors associated with the self-management of individuals living with one or more chronic, non-communicable diseases (NCDs) in South Asian settings. While I appreciate the authors’ responsiveness to our feedback to re-run the literature search and to update the language in the manuscript, I have a few questions and hope this can be clarified: | |
| Table 2: what does it mean if there are no subscripts next to the arrow? Are there 0 self-management behaviors observed for a particular structural or intermediary-level determinant? It is difficult to understand why this information is highlighted, especially with the very brief mention of Table 2 in line 183. I’d appreciate additional details either in the footnote and/or in the quantitative synthesis of the results section. | Thank you for highlighting this issue.  We have now updated table 2. For clarity, the text in the third row (below “type of NCD”) has been changed to “observed self-management behaviour” (previously “dependent variable”). We have provided additional clarification in the table footnote that this could either be singular or multiple self-management behaviour/s, or self-management observed using a composite scale. We have also clarified in the footnotes that for studies observing multiple self-managment behaviours, the subscripts placed alongside the arrows indicate the number of behaviours for which associations were reported. All changes are highlighted in the marked version of the resubmitted manuscript. |
| While I appreciate the effort to better define the concepts of socioeconomic position and SES in your revision, it is still not clear to me. Especially in Table 2, it’s a bit confusing to see education as a separate category when it can be encompassed within socioeconomic status. | Thank you for raising this point.  We agree that measures of socio-economic status includes an assessment of the level of education. We have carefully reviewed the extracted data in light of your comment. In the majority of studies (9 out of 11), observations reported under “socio-economic status” actually measured only individual or household income. On the other hand, two studies have used the kuppuswamy SES scale which takes education into account.  To reflect more accurately that the majority of studies refer to individual/household income rather than SES, we have changed the wording from “socio-economic status” to “individual/household Income” in both table 2 as well as throughout the manuscript text. We have also clarified in table 2 footnotes, and the results section that 2 of these studies used a SES scale which takes education into account. See highlighted text from the results section:  “Higher individual or household income was associated with better self-management in six studies, of which four were moderate-quality studies. A limited set of studies, including two that observed wider socio-economic status (incorporating occupation and education in addition to household income) and observing multiple self-management behaviours reported variations by the type of observed behaviour, with poorer dietary and exercise behaviours reported in higher SES categories” |
| It is interesting that some countries have 0 studies published and included in this review, with the updated literature search—why is this not acknowledged in the manuscript as part of the discussion? What’s the generalizability of the findings and are there limitations to providing a ‘South Asian perspective’ (as mentioned in lines 82 and 406)? | Thank you for highlighting this important point. We have acknowledged this limitation at the very beginning of the strengths and limitations section. See highlighted text from the discussion section:    “Firstly, we aimed to identify studies across all South-Asian countries, but despite a comprehensive search we were limited to studies conducted in four South Asian countries (Bangladesh, India, Nepal and Pakistan) only. Our findings may thus may have limited generalisability to other South Asian countries which were not represented.” |
| There are an admixture of race and ethnicities living in each South Asian country setting (as defined by the World Bank definition). Considering that racism exists in healthcare and could influence self-management behaviors—could this be included in your Figure 2 conceptual model? | Thank you for the valuable comment. We agree with the point raised by the reviewer and have updated the conceptual model accordingly to include racism in healthcare settings (Figure 2) |
| Line 51: Is it appropriate to only mention WHO protocols if the World Bank definition for South Asia countries was used to define the countries included in this review? There is no context for this information, and it is not clear to me why this remains a main discussion point. | Thank you for pointing this out. We have rephrased the text in line 51 to provide a more accurate summary of the discussion, relevant to the context of the review .  “Multi-component, whole-systems approaches could boost self-management in commonly occurring NCDs. Key areas include empowerment and education of patients and the wider community, design and delivery of bespoke behavioural interventions and a stronger emphasis on supporting self-management in healthcare settings.” |
